# Pregnancy Desire, Partner Serodiscordance, and Partner HIV Disclosure among Reproductive Age HIV-Infected Women in an Urban Clinic

**DOI:** 10.1155/2016/8048457

**Published:** 2016-05-26

**Authors:** Corinne M. Rhodes, Susan Cu-Uvin, Aadia I. Rana

**Affiliations:** ^1^General Internal Medicine, Massachusetts General Hospital, Harvard Medical School, Boston, MA 02114, USA; ^2^General Internal Medicine, Alpert Medical School of Brown University, Rhode Island Hospital, Providence, RI 02903, USA; ^3^Department of Medicine, Alpert Medical School of Brown University, The Miriam Hospital, Providence, RI 02906, USA

## Abstract

Women comprise 25% of the US HIV epidemic, with many women of reproductive age. There is a need for providers to address the reproductive needs and desires of women with HIV given that effective antiretroviral therapy has transformed HIV into a chronic disease. This cross-sectional study shows high rates of partner serodiscordance (61%) and moderate HIV disclosure to partners (61%). Patients surveyed reported practitioners discuss condoms (94%) and contraception (71%) more often than pregnancy desire (38%). In our sample, 44% of the surveyed women intended future pregnancy, whereas women who did not intend future pregnancy cited HIV/health and serodiscordance as the most common reasons (56% and 35%, resp.). There was no difference in the knowledge of mother-to-child transmission risk between women who intended or did not intend future pregnancy (*p* = 0.71). These results underline the need for provider training in reproductive counseling to promote risk reduction and education.

## 1. Introduction

There are 280,000 HIV-infected women and 200,000 heterosexual serodiscordant couples living in the United States, many of whom need reproductive health services [1, 2]. Cross-sectional US studies report varied rates of pregnancy desire by women across time: from 29% in a nationally representative survey in 1998 [3] to 59% in an urban sample in 2010 [4]. A national sample of HIV-infected women from 2007 to 2009 [5] showed high rates (86.4%) of unintentional pregnancy, suggesting that pregnancy counseling should extend to all HIV-infected women regardless of stated desire of future pregnancies.

According to recent US data, over half of the HIV-infected women are in serodiscordant relationships with similar reproductive desires to HIV-negative women [5–7]. One-third of women deny any discussion about reproductive health with their HIV provider [5, 7, 8]. This highlights the need for HIV providers to discuss strategies to reduce HIV transmission including ensuring HIV suppression with antiretroviral therapy (ART), timed conception, and preexposure prophylaxis (PrEP) [4].

We seek to examine rates and impact of partner serodiscordance, partner HIV disclosure, practitioners' reproductive counseling, and mother-to-child transmission (MTCT) knowledge on HIV-infected women's reproductive desires at a large urban HIV clinic in New England.

## 2. Methods

A convenience sample of 100 English speaking adult HIV-infected women of reproductive age were enrolled from March 2012 to March 2014 at the Miriam Hospital Immunology Center, a large urban academic clinic serving 1600 HIV-infected patients in Rhode Island. Women were recruited consecutively by study personnel at clinic visits to complete an administered survey and were provided with a $10 retail gift card as an incentive. The survey is available as a supplemental file (Supplementary Material is available online at http://dx.doi.org/10.1155/2016/8048457). Exclusion criteria included current pregnancy, menopause (absent menses >1 year), or hysterectomy. The Miriam Hospital Institutional Review Board approved all study procedures.

Variables surveyed included self-reported demographics and clinical characteristics including ART use, sexual history (including condom and contraceptive use), sexual partners and HIV disclosure, self-defined HIV symptoms as either none, mild, moderate, or severe, and desire for future pregnancy. Women reported reasons for presence or absence of future reproductive desire as well as the effect of HIV, CD4 cell count/viral load (VL), and partner serodiscordance on their desire for future pregnancy. Participants reported previous discussions with providers about condom use, contraception, and desire for future pregnancy. Providers at the clinic include either internal medicine or obstetrics/gynecology doctors. Participants were also asked a multiple-choice knowledge-based question about MTCT. CD4 count was collected by chart abstraction.

Results are reported using proportions, means, and standard deviations. Answer to “*Do you desire to have more children in the future?*” determined future reproductive desire. Continuous data were analyzed using two-tailed Student's *t*-tests and categorical data with Fisher's exact/chi squared tests with significance set at 0.05%. Multivariable logistic regression was conducted using purposeful selection [6] with desire for future pregnancy as dependent variable by including candidates with *p* values <0.25 (identified by univariate screen), sequentially removing nonconfounding variables with *p* > 0.10, and testing each noncandidate for confounding. Results are reported in adjusted odds ratios (AOR) with 95% confidence intervals (CI). We used SAS version 9.4 (SAS Institute Inc., Cary, NC) for statistical analyses.

## 3. Results

Of the 102 women who met inclusion criteria, 2 declined participation, citing time constraints. The mean age of this cohort was 36.7, and self-reported race was 32% white, 27% black, 22% Hispanic, and 19% multiple race, Asians, American Indians, or others.  Table 1 compares demographic and clinical information in groups with and without desire for future pregnancy. Women desiring pregnancy were younger (32.6 versus 39.9, *p* < 0.0001) and reported less prior pregnancies (2.9 versus 4.0, *p* = 0.02) and deliveries (1.9 versus 2.8, *p* = 0.01). Other clinical markers including CD4 cell count, self-reported HIV symptoms, and AIDS diagnosis were not statistically different.

Table 2 reports sexual history, fertility desire, and provider communication. Most women in the study were sexually active (desire 64% versus no desire 74%, *p* = 0.38). There were high but similar rates of serodiscordant partnerships among sexually active women (desire 69% versus no desire 71%, *p* = 0.77) with both groups reporting that concern of infecting partner with HIV affected pregnancy plans (desire 51% versus no desire 33%, *p* = 0.12). Despite these concerns, only 20% of women with reproductive intent were referred to a reproductive specialist to discuss alternative methods of pregnancy.

Nondisclosure of HIV status to any partner in the prior year was relatively high (no desire 29% [*n* = 14] versus desire 18% [*n* = 7], *p* = 0.53). Condom use rates and type of birth control in women with or without reproductive desire did not differ (Table 2).

Of 44 women with desire for future pregnancy, 84% thought they would have a future child and 10 women (23%) currently reported condomless sex with the goal of pregnancy. Interestingly, two women (4%) who did not report desire for a future pregnancy reported that they thought they would have a future child. Just under half of HIV-infected women reported that their HIV diagnosis impacted their pregnancy plans regardless of their desire for future pregnancy (45% versus 45%, *p* = 1). There was no difference (*p* = 0.71) in the knowledge-based MTCT question: “*If you were to become pregnant, what is the probability that your child will contract HIV with proper medication and medical guidance?*”

Provider rates of discussion were the highest for condom use (desire 96% versus no desire 93%, *p* = 0.69) followed by contraception use (desire 64% and no desire 77%, *p* = 0.19) and the lowest in assessing desire for pregnancy (desire 41% versus no desire 35%, *p* = 0.68). Women desiring pregnancy were more likely to initiate a discussion with providers regarding pregnancy intent (42% versus 5%, *p* ≤ 0.0001).

**Figure 1:**
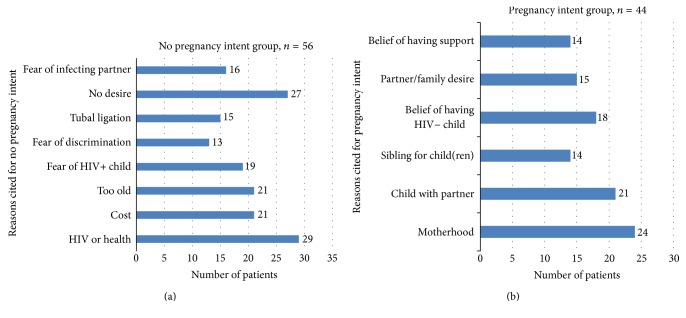
(a) Reasons for not desiring future pregnancy and (b) reasons for desiring future pregnancy.

Figure 1(a) highlights the reason(s) why women reported that they do not desire future children: HIV/health (52%), no desire for children (48%), cost (38%), “too old” (38%), and fear of an HIV positive child (34%).  Figure 1(b) reports the most common reason(s) to desire a future child: “experiencing motherhood” (55%), wanting a child with the current partner (48%), belief that the child will be HIV negative (41%), and partner/family desire for child (34%).

Logistic model results identified crude univariate predictors for desire of future pregnancy. Factors that reduced desire of future pregnancy include age (OR: 0.84, 95% CI: 0.77–0.90), number of prior pregnancies (OR: 0.82, 95% CI: 0.68–0.98), current children (OR: 0.26, 95% CI: 0.08–0.81), and time since HIV diagnosis (OR: 0.93, 95% CI: 0.87–0.99). Both current ART regimen (OR: 3.8, 95% CI: 1.1–13.2) and partner desire for child (OR: 32.2, 95% CI: 9.4–110.1) increased likelihood of desiring future pregnancy. Multivariate model significant predictors were age (AOR: 0.78, 95% CI: 0.68–0.90) and partner's desire for child (AOR: 45.3, 95% CI: 8.6–238.0).

## 4. Discussion

Our study sample indicated that 44% of the reproductive aged HIV-infected women desire future pregnancy, 84% of whom think they will become pregnant. A majority of our sample reported serodiscordant relationships, and 21% reported not sharing HIV status with any sexual partner in the previous year. Women reported discussions with providers about condoms (94%) and contraception (71%) but discussed current or future specific reproductive desires less often (38%). The reported lack of provider counseling for safe pregnancy planning could lead a patient to believe either that the provider has bias against pregnancy or that the patient should not become pregnant.

Our reported rate of reproductive desire is consistent with recent US studies [4, 6, 7] but higher than earlier surveys [3]. This may be due to improvements in HIV treatment throughout the past decade. The rate of serodiscordance in the present study is higher than previously reported [3, 10] and notable as 38% of women reported that fear of infecting partner affected plans to pursue pregnancy. This highlights an opportunity for increased counseling on risk-reduction strategies among HIV-infected women. In our sample, 10% of all women reported current condomless sex with the goal of pregnancy and only 20% of women desiring future pregnancy reported referral to a reproductive specialist. This indicates the need for education about safer serodiscordant partner conception options. Recent surveys [11, 12] showed provider variability in awareness of and referrals for PrEP, timed unprotected intercourse, intrauterine insemination (IUI), and in vitro fertilization (IVF) highlighting the need for provider training on these issues.

Our final multivariate model included age and partner desire for children as significant predictors of desire for future pregnancies. There are a variety of significant predictors in previous models, with age being the most consistent [3, 4, 13, 14]. Number of children, length of time since diagnosis, and ART use were significant in a few models, but we presume these terms are collinear with age in our model. Partner's desire for children is a strong influence on reproductive desires of women in the US [4, 14] and significant in our model. This multivariate analysis is significant as it suggests that HIV-infected women are making decisions regarding future pregnancies based on similar factors to HIV-negative women.

Limitations of this study include a small sample size and cross-sectional study design which is not able to capture evolving trends in pregnancy desire among HIV-infected women. However, we were able to include a diverse representation of race and age in this cohort and did attempt to capture both prior and future intent of pregnancy among HIV-infected women. Recruitment of women in their forties could be viewed as a limitation when generalizing to a younger cohort; however, 18% of women above forty reported desire for pregnancy and contributed to descriptive endpoints.

## 5. Conclusions

It is important that each HIV-infected woman receive comprehensive reproductive health counseling, including assessment of pregnancy desire with defined discussions on reproductive intent. These conversations can be an opportunity to identify serodiscordant couples, assess disclosure of HIV status to sexual partners, and initiate education regarding safer options to avoid or reduce partner and vertical transmission such as PrEP, timed unprotected intercourse, IUI, and IVF. Incorporating training modules for providers managing reproductive aged HIV-infected women's HIV and overall primary care could improve counseling rates and reduce HIV transmission risk.

## Supplementary Material

Supplementary material includes the pregnancy survey that was administered to women meeting inclusion and lacking exclusion criteria by trained research assistants after consent of patients. The survey was developed after reviewing the prior literature and compiling questions of interest.

## Figures and Tables

**Table 1:** Demographics and clinical information for women with HIV of reproductive age.

		No reproductive desire, *n* = 56	Desire for future children, *n* = 44	*p* value
Age: mean (SD)	Age at enrollment	39.9 (5.2)	32.6 (7.1)	<0.0001

Self-reported race	White	21 (38%)	11 (25%)	0.35
	Black	12 (21%)	15 (34%)	
	Hispanic	11 (20%)	11 (25%)	
	Other or multiple races	12 (21%)	7 (16%)	

CD4: mean (SD)	Most recent	588 (311)	586 (289)	0.97

AIDS	Self-reported AIDS	11 (20%)	6 (14%)	0.44

ART status	Yes	52 (93%)	34 (81%)	0.13
	No, not indicated^*∗*^	3 (5%)	3 (7%)	
	No, but indicated	1 (2%)	5 (12%)	

Self-reported HIV symptoms	None	35 (65%)	31 (70%)	0.17
	Mild	12 (22%)	4 (9%)	
	Moderate	7 (13%)	9 (20%)	
	Severe	0	0	

Parity: binary	Currently having child	51 (91%)	32 (73%)	0.03

Parity: mean (SD)	Pregnancies	4.0 (2.6)	2.9 (2.2)	0.02
	Deliveries	2.8 (2.3)	1.9 (1.4)	0.01
	Abortions	20 (36%)	16 (36%)	1

^*∗*^ART indications at the time of data collection: CD4 count < 500, AIDS defining illness, pregnancy, acute opportunistic infections, *Mycobacterium tuberculosis*, HIV-associated nephropathy, and Hepatitis B coinfection when treatment is indicated [9].

**Table 2:** Sexual history, fertility desires, and provider communication for women with HIV of reproductive age.

		No reproductive desire, *n* = 56	Desire for future children, *n* = 44	*p* value
^*∗*^Sexual activity	Sexually active	36 (64%)	32 (74%)	0.38
	Not sexually active	20 (36%)	11 (26%)	

^*∗∗*^Partner's HIV status	Known: HIV negative	34 (71%)	27 (69%)	0.77
	Known: HIV positive	10 (21%)	10 (26%)	
	Unknown	4 (8%)	2 (5%)	

^*∗*^Shared HIV status with partner in the last year	All	28 (57%)	26 (67%)	0.53
	Some	7 (14%)	6 (15%)	
	None	14 (29%)	7 (18%)	

^*∗*^Condom use	100%	33 (59%)	20 (48%)	0.11
	75–99%	6 (11%)	5 (12%)	
	50–74%	3 (9%)	0	
	25–49%	2 (4%)	3 (7%)	
	<25%	10 (18%)	14 (33%)	

Birth control method(s)	Barrier	32 (57%)	20 (45%)	0.31
	Permanent	15 (27%)	6 (14%)	0.14
	None/abstinence/natural	18 (32%)	21 (48%)	0.15
	Hormonal contraceptive/long acting reversible contraception	10 (18%)	10 (23%)	0.62

Fertility desire	*Do you desire to have children in the future?*	0	44 (100%)	<0.0001
	*Do you think you will have children in the future?*	2 (4%)	37 (84%)	<0.0001
	*Does your partner desire to have more children? *	6 (13%)	29 (83%)	<0.001
	*Are you currently having unprotected sex with the goal of becoming pregnant?*	0	10 (23%)	<0.0001
	*Has your HIV diagnosis impacted your plans regarding pursuing pregnancy?*	25 (45%)	20 (45%)	1
	*Have improvements in treatment and intervention made you desire more children?*	6 (11%)	21 (48%)	<0.0001
	*Does your viral load or CD4 count affect your plans to pursue pregnancy?*	7 (13%)	8 (18%)	0.39
	^*∗∗∗*^ *Does your concern of infecting your (HIV negative) partner with HIV affect your plans to pursue pregnancy?*	16 (33%)	19 (51%)	0.12
	*Have you considered or used alternative options (not unprotected sex) to pursue pregnancy?*	4 (7%)	8 (20%)	0.11

^*∗*^MTCT rate	<1	30 (55%)	29 (66%)	0.71
	5–10%	11 (20%)	7 (16%)	
	10–20%	3 (5%)	1 (2%)	
	>25%	11 (20%)	7 (16%)	

Healthcare provider communication	*Has your medical provider talked to you about whether you were interested in becoming pregnant?*	20 (36%)	18 (41%)	0.68
	*Have you expressed an interest in becoming pregnant to your medical provider?*	3 (5%)	19 (43%)	<0.0001
	* Has your medical provider talked to you about birth control or contraception?*	43 (77%)	28 (64%)	0.19
	* Has your medical provider talked to you about condoms to prevent STDs and HIV transmission?*	52 (93%)	42 (96%)	0.69
	*Has your medical provider referred you to a reproductive specialist to discuss methods to pursue pregnancy?*	4 (7%)	9 (20%)	0.07

^*∗*^ *n* = 99 due to omission by one respondent.

^*∗∗*^ *n* = 87 excluding patients with no sexual partner in the prior year.

^*∗∗∗*^ *n* = 85 excluding patients with no sexual partner in the prior year and 2 omissions.
