# Supplementary material for: Pregnancy Desire, Partner Serodiscordance, and Partner HIV Disclosure among Reproductive Age HIV-Infected Women in an Urban Clinic
Source: Infect Dis Obstet Gynecol. 2016 May 26;2016:8048457. doi: 10.1155/2016/8048457 (PMC4899585; doi:10.1155/2016/8048457)
Supplement: Supplementary file 1 — Supplementary material includes the pregnancy survey that was administered to women meeting inclusion and lacking exclusion criteria by trained research assistants after consent of patients. The survey was developed after reviewing the prior literature and compiling questions of interest. [file 8048457.f1.docx]

Questionnaire for HIV positive women of reproductive age

**BACKGROUND**

1. How old are you? ______

2. Are you Hispanic or Latina?

☐ YES ☐ NO

3. What is your racial background? Check all that apply.

☐ White ☐ Black or African American

☐ Asian ☐ Native Hawaiian or Pacific Islander

☐ American Indian or Alaska Native ☐ Other__________________

4. What is your relationship status? Check all that apply.

☐ Married

☐ In committed relationship

☐ Dating, not in a committed relationship

☐ Not currently dating

5. What is your current sexual activity status?

☐ Sexually active with one partner

☐ Sexually active with multiple partners

☐ Not currently sexually active

6. How many times have you been pregnant? (please circle)

1 2 3 4 5 6 7 8 9 Other ______

7. How many children have you delivered? (please circle)

1 2 3 4 5 6 7 8 9 Other ______

8. How many terminations or abortions have you had? (please circle)

1 2 3 4 5 6 7 8 9 Other ______

9. How many miscarriages have you had? _____ (please circle)

1 2 3 4 5 6 7 8 9 Other ______

10. How old were you when you were told you had HIV? _____

11. Have you ever injected drugs?

☐ YES ☐ NO

12. Does your current sexual partner(s) have HIV?

☐ YES

☐ NO

☐ DON’T KNOW

13. In the past year (please check the ONE statement which most applies to you)

☐ ALL of my sexual partner(s) knew about my HIV infection

☐ Some of my sexual partner(s) knew about my HIV infection

☐ NONE of my sexual partner(s) knew about my HIV infection

14. How often do you use condoms with your sexual partner(s)?

☐ 100%

☐ 75-99%

☐ 50-74%

☐ 25-49%

☐ <25%

15. What is your primary method of birth control? Check all that apply.

☐ continuous abstinence

☐ natural family planning/rhythm method

☐ barrier methods (male or female condoms, contraceptive sponge, diaphragm, cervical cap, cervical shield)

☐ hormonal methods (oral contraceptives, the patch, shot/injection, vaginal ring)

☐ implantable device (implantable rod, intrauterine device)

☐ permanent birth control methods (sterilization implant, surgical sterilization)

☐ emergency contraception

☐ no current birth control methods

**HEALTHCARE**

1. Has your medical provider talked to you about whether you were interested in becoming pregnant?

☐ YES ☐ NO

2. Have you expressed an interest in becoming pregnant to your medical provider?

☐ YES ☐ NO

3. Has your medical provider talked to you about birth control or contraception?

☐ YES ☐ NO

4. Has your medical provider talked to you about condoms to prevent STDs and HIV transmission? ☐ YES ☐ NO

5. Has your medical provider referred you to a reproductive specialist to discuss methods to pursue pregnancy? ☐ YES ☐ NO

**CLINICAL DETAILS**

1. How would you classify your current HIV symptoms?

☐ None ☐ Mild

☐ Moderate ☐ Severe

2. List most recent CD4 count ______

3. Have you ever been diagnosed with AIDS (CD4 count <200 or AIDS defining illness)?

☐ YES ☐ NO

4. Do you currently take any medicines to treat your HIV infection (antiretroviral medicines)?

☐ YES ☐ NO

4A. If you are not on antiretrovirals, does your provider want you to take them?

☐ YES ☐ No

5. Do you currently have any other sexually transmitted diseases?

☐ YES ☐ NO

6. Have you undergone a hysterectomy?

☐ YES ☐ NO

7. Have you undergone bilateral tubal ligation?

☐ YES ☐ NO

8. Have you gone through menopause (no menses in the last year)?

☐ YES ☐ NO

**FERTILITY INTENTIONS**

1. Do you desire to have children in the future?

☐ YES ☐ NO

a. If yes, what is your primary reason? Check all that apply.

☐ I want to experience motherhood

☐ I want to have a child with my current partner

☐ I want a sibling for my other child(ren)

☐ I believe I will have an HIV negative child

☐ My partner or family wants me to

☐ I believe I will have support raising my child

b. If no, what is your primary reason? Check all that apply.

☐ HIV or health reasons

☐ I think I am too old

☐ I cannot afford more children

☐ I fear my child will be infected

☐ I am concerned about exposing my child to discrimination just because I am HIV positive

☐ I have had tubal ligation

☐ I don’t want any (more) children

☐ I am afraid of infecting my partner

2. If you have previously had a child while HIV positive, did you or your child experience any discrimination in the community (school, work, doctor’s office, church, within the family, etc.) due to your HIV status?

☐ YES

Where? __________________________________

By Whom? ________________________________

☐ NO, we have not disclosed my HIV status to anyone

☐ NO, we have not experienced any discrimination

3. Do think you will have children in the future?

☐ YES ☐ NO

4. Are you currently trying to get pregnant?

☐ YES ☐ NO

5. Are you currently having unprotected sex with the goal of becoming pregnant?

☐ YES ☐ NO

6. Before you were diagnosed with HIV, did you plan to have children?

☐ YES ☐ NO

7. Has your HIV diagnosis impacted your plans regarding pursuing pregnancy?

☐ YES ☐ NO

8. Does your viral load or CD4 count affect your plans to pursue pregnancy?

☐ YES

☐ NO

☐ DON’T KNOW

9. Have improvements in treatment and intervention made you desire more children?

☐ YES

☐ NO

☐ DON’T KNOW

10. Has your HIV diagnosis made you want children earlier than you previously would have planned?

☐ YES ☐ NO

11. Have you considered or used alternative options (not unprotected sex) to pursue pregnancy?

☐ YES

☐ NO

☐ DON’T KNOW

12. Does the cost of alternate options to pursue pregnancy effect your use of them?

☐ YES ☐ NO

13. Does your partner want to have more children?

☐ YES

☐ NO

☐ DON’T KNOW

14. Does your concern of infecting your partner with HIV effect you plans to pursue pregnancy?

☐ YES

☐ NO

☐ DON’T KNOW

15. If you were to become pregnant, what is the probability that your child will contract HIV with proper medication and medical guidance? (check one)

☐ <1%

☐ 5-10%

☐ 10-20%

☐ >25%
